# Unintentional Drug-related Deaths in Cambridgeshire: A Retrospective Observational Study

**DOI:** 10.7759/cureus.6750

**Published:** 2020-01-23

**Authors:** Tahir S Khan, Adrian Boyle, Susie Talbot

**Affiliations:** 1 School of Clinical Medicine, University of Cambridge, Cambridge, GBR; 2 Emergency Medicine, Addenbrookes Hospital Cambridge University, Cambridge, GBR; 3 Public Health Directorate, Cambridgeshire County Council, Cambridge, GBR

**Keywords:** drugs-related deaths, overdose, prescription drugs, illicit drugs, risk factors

## Abstract

Background: Drug-related deaths are a growing public health problem in the United Kingdom, overtaking road fatalities and homicides in terms of annual deaths. In this study, we investigated the causes and circumstances of unintentional drug-related deaths occurring in the county of Cambridgeshire, with the objective of identifying the prevalence of physical, mental, and social health problems within this cohort.

Methods: We collected data on the demographics and mental and physical health of, and drugs contributing to, 30 consecutive unintentional drug-related deaths recorded by the Cambridgeshire and Peterborough County Council Coroners in 2017. A retrospective observational study was used, and data were collected by manual extraction from coroners’ files.

Results: Social isolation was identified as a recurring theme amongst the decedents, although homelessness was found in very few cases. Pharmacologically, multiple drug toxicity and opioid toxicity were highly prevalent, whilst prescription opioids were implicated in more cases than heroin. Chronic pain was also highly prevalent amongst the decedents, and a history of mental illness was found to occur in the majority of cases.

Discussion: Our findings show that reports from the coronial system provide a rich narrative to understand the causes of drug-related deaths. We have identified that individuals who die from drug-related deaths frequently have multiple adverse physical, mental, and social problems. This implies that any attempt to reduce drug-related deaths requires a multi-faceted and multi-disciplinary approach.

## Introduction

Drug-related death is the fifth most common preventable cause of death in the United Kingdom, after neoplasms, cardiovascular diseases, injuries, and respiratory diseases [1]. In 2017 alone, there were 2,779 deaths related to accidental drug poisoning occurring in England and Wales [2]. These are the highest figures since the beginning of data collection, and, by comparison, there were 5,821 suicides, 1,793 road fatalities, and 709 homicides in England and Wales in the same year [3-5]. It is apparent that drug-related deaths are a major public health issue.

The last two decades have seen a worldwide trend of decreasing deaths from illicit drug use and increasing deaths from prescription drug use [6]. Cocaine, prescription opioids, and heroin are the drugs most commonly involved in unintentional drug overdoses worldwide, whilst several studies have found tricyclic antidepressants to be second only to opioid analgesics as the commonest prescription drug taken in fatal drug overdose [6-7]. The USA is considered to be at the center of a prescription drug abuse crisis, having seen an alarming rise in the rate of opioid prescriptions coupled with a three-fold increase in deaths involving prescription opioids [8-9]. In the UK, deaths involving prescribed benzodiazepines, gabapentin, and pregabalin have shown an increase in the last four years, following the worldwide trend of increasing prescription drug abuse [10-11].

Whilst previous literature has looked at drug-related deaths largely from a viewpoint of the temporal trends and substances involved, there are fewer studies that have looked at the relationship of factors such as physical and mental illness to drug-related deaths. This may usefully guide policies and prevention efforts. In the USA, studies to date have found that those who have mental health disorders are more likely to suffer from drug abuse or drug dependence [12]. Furthermore, histories of mental illnesses and chronic pain have been found in a large proportion of prescription drug overdose deaths in the USA [13-14]. However, along with there being almost no data on the impact of these factors on drug-related deaths in the UK, there is also limited literature covering the relationship between illicit drug-related deaths and mental and physical illness.

Subsequently, we carried out an extensive analysis of a small group of unintentional drug-related death victims in the county of Cambridgeshire. Our methodology of using coroner’s reports provided a detailed narrative from multiple sources about the circumstances leading to each death. This allowed us to achieve our aims of describing the social, pharmacological, and health-related risk factors for unintentional drug-related deaths in this area. In addition, we also reported the involvement of local social and healthcare services in each case to look for any limitations in their provisions.

## Materials and methods

Case definition

The coroner’s inquest verdict was used to identify cases. Drug-related death was defined according to the current Office for National Statistics definition of "drug misuse death," which include:

· deaths where the underlying cause is drug abuse or drug dependence,

· deaths where the underlying cause is drug poisoning and where any of the substances controlled under the Misuse of Drugs Act 1971 are involved [2].

This excludes poisonings by noxious substances (such as carbon monoxide) and chemicals other than drugs and medicaments, as well as deaths related solely to alcohol. In addition, we excluded drug-related suicidal deaths as we wanted the scope of our study to concentrate specifically on unintentional drug-related deaths and how these may be prevented.

Study sample 

The study sample was taken from a manual review of the coroner’s reports at Cambridgeshire and Peterborough Coroner’s Office. All deaths registered in 2017 which mentioned drugs (e.g., "opiate toxicity," "mixed drug toxicity," "chronic drug misuse," etc.) in the cause of death were identified for further review. Initially, those coroner reports that returned with a verdict of "suicide" following the inquest were removed. Subsequently, those files which were "open" for an inquest were also removed, along with those in which the cause of death was not related to drug toxicity (e.g., "hypovolemic arrest"). Thirty cases of unintentional drug-related deaths registered in 2017 in Cambridgeshire were identified.

Study setting

The study setting was the geographical county of Cambridgeshire in the UK, which includes the separately administered area of the City of Peterborough. These areas have a combined population of 847,151, with an equal division between males and females [15]. In terms of age distribution, 63% of the population is 16-64 years old, and in terms of deprivation, the county ranks 133 out of 152 upper-tier local authorities in England on the Index of Multiple Deprivation (IMD), with 1 being the most deprived [16].

Data analysis

Analyses were carried out on all cases of unintentional drug-related deaths in our cohort. Demographic characteristics were described, including sex, age, employment status, housing, relationship status, and criminal justice involvement. The cause of death was also noted, along with the drugs present in the toxicology report, and this information was used to report the psychoactive drugs implicated in the deaths. Healthcare information, including any history of physical or mental illnesses, were noted from reports present in the files, mostly from General Practice (GP). Prescribed medications were also noted from the GP letters to ascertain if prescription or non-prescription drugs correlated with toxicology reports. Community services information was then noted for those cases where it was present, including Drug and Alcohol Services and Mental Health Services. Specifically, with regards to mental illness, the following three risk factors for toxic overdose were noted: history of self-harm, suicide attempts, and previous overdoses. Finally, other risk factors for drug abuse mentioned in the files were noted, including criminal justice involvement and domestic abuse.

Missing data 

A few of the cases had incomplete files of coroner’s reports, such that certain data points were missing. This has been accounted for in the data presentation by noting the "unknown" cases for each category where this is relevant.

Confidentiality 

In order to preserve the identity of the deceased individuals, data groups containing less than five individuals have been reported as "<5."

## Results

We retrieved data from coroner’s files for 30 individuals who died from unintentional drug-related death in 2017 in Cambridgeshire.

Demographics

We reported the age, sex, employment status, housing, and past criminal justice involvement of the 30 individuals (Table 1). More than half of the decedents (57%) were 30-49 years old, whilst 63% were male. Half of the decedents were unemployed, in addition to half being single. In terms of housing, there was a roughly equal distribution of subjects who lived alone or who lived with friends/family, whilst less than five subjects lived in supported housing, and less than five subjects were homeless. Finally, 30% of subjects had past involvement with the criminal justice system.

**Table 1:** Demographic characteristics of drug-related deaths in Cambridgeshire and Peterborough, 2017

Demographic characteristic	Groups	Number of deaths (%)
Age (years)	<20	<5 (<17%)
	20-29	5 (17%)
	30-39	7 (23%)
	40-49	10 (33%)
	50-59	<5 (<17%)
	>59	<5 (<17%)
Sex	Male	19 (63%)
	Female	11 (37%)
Employment status	Employed	6 (20%)
	Unemployed	15 (50%)
	Retired/Student	<5 (<17%)
	Unknown	5 (17%)
Housing	Alone	12 (40%)
	With friends/family	13 (43%)
	Supported housing	<5 (<17%)
	None (homeless)	<5 (<17%)
Relationship status	Single	15 (50%)
	In a relationship (not married)	<5 (<17%)
	Married	5 (17%)
	Separated/Divorced	<5 (<17%)
	Unknown	<5 (<17%)
Other	Criminal justice involvement	9 (30%)

Toxicology

We reported the numbers and proportions of psychoactive substances implicated in the deaths investigated (Table 2). Of the 30 deaths, 14 were noted as occurring from opioid toxicity alone, whilst a further 13 occurred due to mixed drug toxicity. Opioids were the most frequently implicated drug class (80%), whilst anti-depressants (43%), cocaine (37%), and benzodiazepines (33%) were also commonly mentioned in toxicology reports. In terms of individual drugs, cocaine was the most commonly mentioned in toxicology reports, followed by heroin (33%) and amitriptyline (27%). A mean of 3.75 drugs per person were implicated in each death.

**Table 2:** All psychoactive drugs implicated in drug-related deaths in Cambridgeshire and Peterborough, 2017

Drug		Number of deaths (%)
Any opioid		24 (80%)
	Heroin	10 (33%)
	Methadone	5 (17%)
	Morphine	5 (17%)
	Codeine	<5 (<17%)
	Dihydrocodeine	<5 (<17%)
	Tramadol	<5 (<17%)
	Fentanyl	<5 (<17%)
Cocaine		11 (37%)
Any amphetamine		<5 (<17%)
	Amphetamine	<5 (<17%)
	MDMA	<5 (<17%)
Any benzodiazepine		10 (33%)
	Diazepam	5 (17%)
Gabapentin		<5 (<17%)
Pregabalin		<5 (<17%)
Nonbenzodiazepines		<5 (<17%)
Alcohol		<5 (<17%)
Cannabis		<5 (<17%)
Anti-depressants		13 (43%)
	Amitriptyline	8 (27%)

We also reported the number of psychoactive prescription drugs found in the toxicology reports (Table 3). Sixteen deaths (53%) involved psychoactive prescription drugs, with 11 of these implicating multiple prescribed psychoactive drugs. Prescription antidepressants were the most commonly implicated (40%), followed by opioids (37%) and benzodiazepines (20%). In terms of individual drugs, amitriptyline was implicated the most (23%), followed by methadone (<17%) and gabapentin (<17%). Out of the 11 individuals whose deaths implicated prescription opioids, eight had been prescribed it for chronic pain, whilst the other three were on methadone prescriptions for opiate substitution treatment.

**Table 3:** Psychoactive prescription drugs mentioned in drug-related deaths in Cambridgeshire and Peterborough, 2017

Prescription drug		Number of deaths (%)
Any opioid		11 (37%)
	Morphine	<5 (<17%)
	Methadone	<5 (<17%)
	Codeine	<5 (<17%)
	Dihydrocodeine	<5 (<17%)
	Tramadol	<5 (<17%)
	Fentanyl	<5 (<17%)
Anti-depressants		12 (40%)
	Amitriptyline	7 (23%)
Any benzodiazepine		6 (20%)
	Diazepam	<5 (<17%)
Pregabalin		<5 (<17%)
Gabapentin		<5 (<17%)
Nonbenzodiazepines		<5 (<17%)

Co-morbidities

The prevalence of health conditions found in the decedents was recorded (Table 4). The majority of the decedents (83%) had a physical illness, whilst more than half of them (53%) had a mental illness. Chronic pain (40%), drug and alcohol dependence (23%), and hepatitis C (17%) were the most common physical illnesses found, whilst depression (23%) and anxiety (<17%) were the most common mental illness found. Out of the nine subjects suffering from any type of chronic pain, seven were prescribed opioids for this, and all seven had these drugs implicated in their death. In addition, we also reported that a significant proportion of subjects had a history of previous overdoses (33%), suicide attempts (23%), and self-harm (17%). Furthermore, several subjects (<17%) had a history of suffering from domestic abuse, and all of these subjects were female.

**Table 4:** Co-morbidities found in drug-related deaths in Cambridgeshire and Peterborough, 2017

Health conditions		Number of deaths (%)
Any physical illness		25 (83%)
	Chronic back pain	5 (17%)
	Chronic leg pain	<5 (<17%)
	Fibromyalgia	<5 (<17%)
	Epilepsy	<5 (<17%)
	Hepatitis C	5 (17%)
	Drug/alcohol dependency	7 (23%)
Any mental illness		16 (53%)
	Depression	7 (23%)
	Anxiety	<5 (<17%)
	Emotionally Unstable Personality Disorder	<5 (<17%)
	Schizophrenia	<5 (<17%)
	Post-traumatic stress disorder	<5 (<17%)

Social services involvement

The involvement of community services in the cases analyzed was also reported (Table 5). 31% of those subjects with a mental illness were unknown to services, and 44% of problematic alcohol users had no history of detox.

**Table 5:** Community services involvement, drug-related deaths, Cambridgeshire and Peterborough, 2017

Condition	Groups	Number of subjects (% of subjects with condition)
Mental illness (n=16)	Known to services	11 (69%)
	Unknown to services	5 (31%)
Drug/alcohol misuse (n=17)	Known to services	16 (94%)
	Unknown to services	<5 (<29%)
Heroin use (n= 8)	Issued naloxone	<5 (<63%)
	Accessed needle exchange services	8 (100%)
Problematic alcohol use (n=10)	History of detox	6 (44%)
	No history of detox	<5 (<50%)

## Discussion

Our results indicate that the demographic characteristics of drug-related deaths in this area are consistent with previous studies that identified males, and those aged 30-50 years, as the groups most at risk of licit and illicit drug overdoses [17-18]. Social isolation was a common finding amongst the decedents, with the majority of subjects being single and unemployed, and nearly half of them living alone. The data included very few people who were homeless; an unexpected finding in light of previous research that has demonstrated that drug and alcohol dependence is common amongst homeless populations [19]. However, this finding may be reflective of the relative socio-economic affluence of the study setting resulting in a smaller homeless population than in other areas.

The drugs implicated in the deaths in this study were also consistent with recent worldwide trends of increasing fatalities from prescription medications with decreasing fatalities from illicit drugs. Prescription opioids were implicated in more deaths than heroin, and methadone was the most common prescription opioid implicated. This mirrors national trends of increasing deaths from prescription opioids and methadone, although the latter may be attributed to an increase in the availability of opioid substitution treatment in the UK [20-21]. Prescribed gabapentin was implicated in as many cases as methadone, whilst prescribed amitriptyline and benzodiazepines were the most highly implicated individual drugs. We also found that the majority of cases involved multiple drug toxicity, with more than a third of cases involving the use of multiple prescription drugs. This indicates that multiple prescriptions of psychoactive drugs are a risk factor for a drug-related death.

Further to this, we also found chronic pain to be the most common physical complaint present in our sample, with more than a third of decedents having a history of chronic pain (back pain, leg pain, fibromyalgia, etc.). The majority of these individuals were on prescription opioids that were implicated in their death, meaning that nearly a quarter of all drug-related deaths in this study implicated prescription opioids that were prescribed for chronic pain. This finding comes at a time when several studies and reviews have questioned the long-term prescribing of opioids for non-malignant pain. A recent review questioned the extended prescription of opioids (>8 weeks), identifying inadequate medical training on pain management and addiction as possible factors contributing to the prescription opioid abuse epidemic [22]. Further study into this field may help ascertain if prescription opioid-related deaths can be reduced by a change in the prescribing practices of opioids for chronic pain at a primary care level.

Finally, mental illness was also very common in people who suffered drug-related deaths in this study, with more than half of the subjects suffering from a mental illness. Whilst previous research has highlighted the link between mental illness and prescription drug overdose deaths, in this study we showed that mental illness remains highly prevalent even when this cohort is extended to include illicit drug-related deaths. Nearly a third of these decedents with mental illnesses were unknown to mental health services, and this underlines the need for involvement of mental health services in tackling drug-related deaths.

Limitations

One possible limitation of this study was that some cases of suicide may have not been identified as such due to the stringent evidence requirements by the law to pronounce suicide. Subsequently, some of our samples may have included suicidal victims who were incorrectly identified as having overdosed unintentionally. Furthermore, since we did not have denominator data, describing a variable as a risk factor was not possible, as this requires high proportions with a variable. Finally, our study was reliant on coroners’ reports for data collection, and these were not always complete, leading to some unknowns in our results.

## Conclusions

Our findings indicate that coronial reports provide a rich narrative to understand the causes of drug-related deaths. We found that social isolation, prescription opioid use, mental illness, and chronic pain are common amongst victims of a drug-related death. We mirrored previous findings of the demographics of drug-related deaths, along with reporting few deaths amongst the homeless population. Multiple drug use was common, and prescription opioids were implicated in more deaths than heroin, with multiple prescription drugs mentioned regularly in toxicology reports. With chronic pain being the most common indication for prescribing opioids, this finding should prompt further research into the treatment of chronic pain and the efficacy of long-term opioid use for this indication. Finally, our study extended previous findings of the high prevalence of mental illness amongst drug-related deaths to show that this pattern continues to occur in a cohort that includes both illicit and licit drug use. Overall, the findings of this study may be used to guide further research into the prevention of drug-related deaths from a public health and primary care perspective.
